# Modulation of gene expression dynamics by co-transcriptional histone methylations

**DOI:** 10.1038/emm.2017.19

**Published:** 2017-04-28

**Authors:** Hyeonju Woo, So Dam Ha, Sung Bae Lee, Stephen Buratowski, TaeSoo Kim

**Affiliations:** 1 Department of Life Science, Ewha Womans University, Seoul, Korea; 2 The Research Center for Cellular Homeostasis, Ewha Womans University, Seoul, Korea; 3 Department of Brain and Cognitive Sciences, DGIST, Daegu, Republic of Korea; 4 Department of Biological Chemistry and Molecular Pharmacology, Harvard Medical School, Boston, MA, USA

## Abstract

Co-transcriptional methylations of histone H3 at lysines 4 and 36, highly conserved methyl marks from yeast to humans, have profound roles in regulation of histone acetylation. These modifications function to recruit and/or activate distinct histone acetyltransferases (HATs) or histone deacetylases (HDACs). Whereas H3K4me3 increases acetylation at promoters via multiple HATs, H3K4me2 targets Set3 HDAC to deacetylate histones in 5′ transcribed regions. In 3′ regions of genes, H3K36me2/3 facilitates deacetylation by Rpd3S HDAC and slows elongation. Despite their important functions in deacetylation, no strong effects on global gene expression have been seen under optimized or laboratory growth conditions. Instead, H3K4me2-Set3 HDAC and Set2-Rpd3S pathways primarily delay the kinetics of messenger RNA (mRNA) and long noncoding RNA (lncRNA) induction upon environmental changes. A majority of mRNA genes regulated by these pathways have an overlapping lncRNA transcription either from an upstream or an antisense promoter. Surprisingly, the distance between mRNA and lncRNA promoters seems to specify the repressive effects of the two pathways. Given that co-transcriptional methylations and acetylation have been linked to many cancers, studying their functions in a dynamic condition or during cancer progression will be much more important and help identify novel genes associated with cancers.

## Introduction

Chromatin is a complex of eukaryotic DNA and histone proteins packaged within the cell and plays essential roles in many cellular processes including RNA polymerase II (RNA polII) transcription, DNA replication, and DNA damage response.^1^ The basic repeating unit of chromatin is the nucleosome consisting of a histone octamer containing four core histones, H3, H4, H2A and H2B and 147 base pairs of DNA.^2^ Nucleosomes directly block transcription by RNA polII and should be evicted or reorganized during RNA polII initiation and elongation.^3^

Post-translational modifications on histone tails including acetylation, methylation, phosphorylation and ubiquitination play important roles in eukaryotic RNA polII transcription.^1, 4^ Histone acetylation can directly promote transcription by disrupting the interaction between DNA and histone proteins. This mark also acts as a binding site for bromodomain proteins associated with factors that regulate chromatin structure and transcription.^1^ Histone acetylation is dynamically regulated by the antagonistic functions of histone acetyltransferases (HATs) and histone deacetylases (HDACs). How HATs and HDACs control histone acetylation at specific regions of a genome remains to be completely understood, but recent studies propose that site-specific histone methylations may affect local histone acetylation by targeting HATs or HDACs or stimulating their activities.^4, 5^ Histone ubiquitination also regulates histone acetylation at a specific genomic position via H3K4 and H3K79 methylation.^6, 7^

Although chromatin factors have been thought to be essential for global gene regulation, a recent study revealed a highly specific effect. Many chromatin regulators had little or no detectable effect on global gene expression in a steady state growth condition.^8^ Instead, single gene studies revealed that many chromatin regulators primarily modulated the kinetics of gene induction without changes in basal or final levels of expression.^9, 10, 11^ Furthermore, loss of chromatin regulators resulted in greater effects on global gene expression during activation or repression upon environmental changes.^12^ Therefore, unlike gene-specific transcriptional activators or repressors turning on or off their target genes, chromatin regulators can function as a transcriptional modulator that controls the kinetics of gene induction or repression. In addition, long noncoding RNA (lncRNA) transcription and lncRNA itself also play critical roles in gene regulation.^13^ Interestingly, ~12% of yeast genes are overlapped with lncRNA transcription.^14^ A majority of these genes tend to be inducible suggesting the importance of overlapping lncRNA transcription in regulation of gene expression dynamics.

In natural growth conditions, cells must reprogram their gene expression patterns to adapt to rapidly changing environmental conditions. Fine tuning of gene expression dynamics by chromatin regulators can coordinate cellular processes to cell adaptation and fitness upon environmental changes. Here we review recent findings on modulation of gene expression dynamics by co-transcriptional methylations, their downstream effectors and overlapping lncRNA transcription.

## Regulation of co-transcriptional methylations of H3K4 and H3K36

Histone methylations can be targeted by multiple distinct mechanisms.^1^ Factors involved in transcription repression contribute to recruitment of repressive histone methyltransferases (HMTs) for H3K9 or H3K27 methylation. In contrast, methylation of histone H3 at lysines 4 or 36 correlates positively with RNA polII transcription rate, and HMTs for these methyl marks interact with elongating RNA polII to localize histone methylations in transcribed regions.^4, 5, 15, 16, 17^

**Figure 1:**
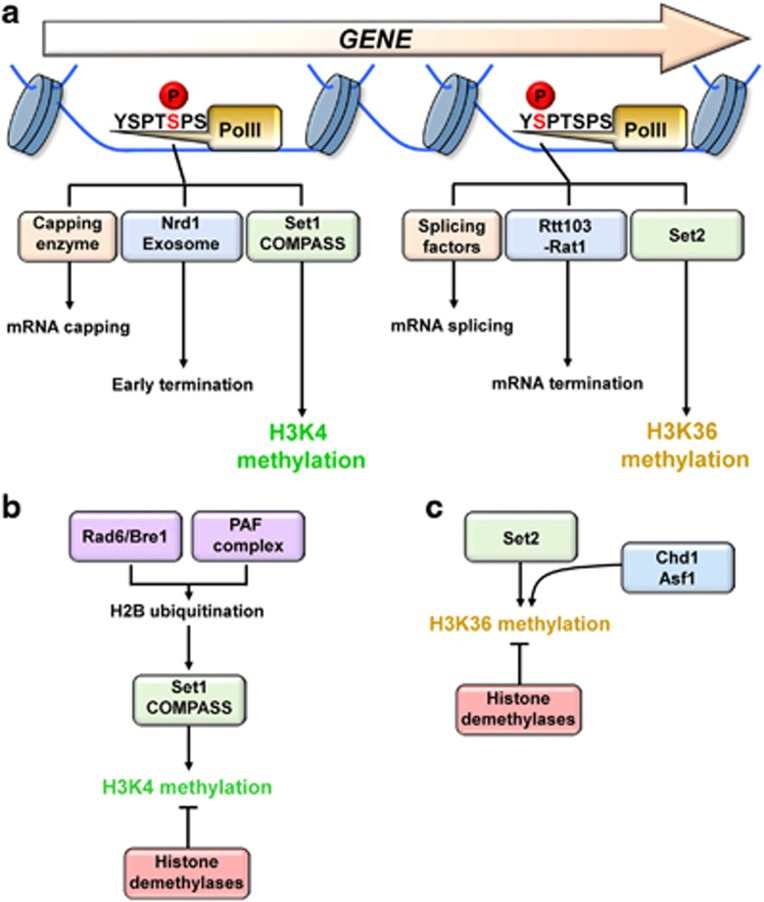
Model for regulation of co-transcriptional methylations. (**a**) At an early stage of transcription, C-terminal domain (CTD) phosphorylation at serine 5 recruits messenger RNA (mRNA) capping machinery and early termination factors. This modification also contributes to recruitment of Set1-COMPASS that methylates histone H3 at lysine 4 in 5′ transcribed regions. During transcription elongation, CTD phosphorylation on serine 2 functions to recruit mRNA splicing machinery and termination factors. In addition, serine 2 phosphorylation of CTD and low levels of serine 5 phosphorylation also create a binding site for Set2 histone methyltransferase (HMT) that methylates H3K36 in 3′ transcribed regions. (**b**, **c**) Factors that influence H3K4 and H3K36 methylation patterns.

During transcription, the C-terminal domain (CTD) of Rpb1, the RNA polII largest subunit, is differentially phosphorylated by at least two kinases (Figure 1a).^18^ Whereas phosphorylation of the CTD repeat heptad sequence on serine 5 by Kin28, a subunit of TFIIH, peaks in transcription start site and 5′ transcribed regions, Ctk1 phosphorylates serine 2 of CTD in 3′ regions.^18^ CTD phosphorylated at serine 5 plays important roles in initiation and early elongation of transcription. This modification mediates a direct interaction between messenger RNA (mRNA) capping enzyme and RNA polII.^18^ Furthermore, transcription termination of snRNAs and short mRNA genes by Nrd1-exosome is stimulated via the interaction between Nrd1 and CTD phosphorylated at serine 5 (Figure 1a). Phosphorylation on serine 2 by Ctk1 also plays important roles during transcription elongation and termination.^18^ Many transcription elongation factors including Spt6 interact with CTD phosphorylated at serine 2 to stimulate elongation. This CTD phosphorylation is also important for co-transcriptional RNA splicing and transcription termination (Figure 1a).^18^

In addition, CTD phosphorylation also generates distinct patterns of histone H3 methylation at lysines 4 and 36 (Figure 1a).^5^ Serine 5 phosphorylation is known to stimulate H3K4 methylation by Set1-COMPASS in 5′ transcribed regions.^19^ Although a direct interaction between phosphorylated CTD at serine 5 and Set1-COMPASS has not been reported yet, inactivation of Kin28 reduced Set1 crosslinking.^19^ In budding yeast, Set1 is the sole methyltransferase for all states of H3K4 methylation. Whereas H3K4me3 and Set1 crosslinking are observed at transcription initiation sites, H3K4me2 and H3K4me1 peak in 5′ and 3′ transcribed regions, respectively.^15, 16, 19, 20^ The factors that affect the gradients of H3K4 methylation need to be determined. It is possible that Set1 activity/stability or composition of COMPASS may be altered during transcription elongation.^21^ Alternatively, Rad6-Bre1 may affect the distribution of H3K4 methylation. H2B ubiquitination on K123 by Rad6-Bre1 is required for H3K4me3 and H3K4me2 (Figure 1b).^7, 22, 23, 24^ Enrichment of H2B ubiquitination in 5′ transcribed regions where H3K4me3 and H3K4me2 peak suggests that dissociation of this complex during elongation may result in peaks of H3K4me1 in 3′ ends of genes.^25^ In higher eukaryotes, multiple H3K4 HMTs including Set1A, Set1B and MLL1-4 proteins have been identified.^26, 27^ Whereas Set1A and Set1B seem to be major HMTs for global H3K4 methylation, MLL3/4 targets H3K4me1 to enhancer regions suggesting the distinct functions of these H3K4 HMTs.^26^

The H3K36 HMT, Set2 preferentially binds to phosphorylated CTD at both serine 5 and serine 2 and targets H3K36me2/3 within the body of genes (Figure 1a).^28, 29, 30, 31^ Mutation in the CTD interacting domain of Set2 or deletion of *CTK1* results in loss of Set2 crosslinking and defects of H3K36me2/3.^29^ Factors involved in transcription elongation also affect H3K36 methylation (Figure 1c). Deletion of *CHD1* chromatin remodeler causes a shift of H3K36me3 to 5′ transcribed regions and Asf1 histone chaperone stimulates H3K36me3 and Set2 crosslinking.^32, 33^ In addition to CTD binding, the N-terminal region of Set2 interacts with histone H4 to stimulate H3K36me2/me3.^34^

Although histone methylation was originally thought to be a permanent mark, enzymes that can reverse histone methylation have been identified. Mammalian lysine-specific demethylase 1 (LSD1) is the first enzyme discovered that demethylates H3K4me2 and H3K4me1, but not H3K4me3.^35^ A second class of demethylases found from bacteria to humans, the Jmjc domain proteins can demethylate all three lysine methylation states and broad ranges of substrates.^36^ Antagonistic functions of HMTs and histone demethylases (HDMs) may maintain optimal levels of histone H3 methylation at lysines 4 or 36 (Figures 1b and c).^37, 38^

## The downstream effectors of co-transcriptional H3 methylations

Unlike acetylation, histone methylation is not known to directly affect chromatin structure. Instead, site-specific methylations act as binding sites for various downstream effector proteins containing chromodomains, tudor domains, PHD fingers and PWWP domains.^1, 4^ These effector proteins are often found in large protein complexes including HATs, HDACs or chromatin remodeling complexes.^6^ The interaction between a site-specific methylation and an effector protein can regulate local chromatin structure and RNA polII transcription.

**Figure 2:**
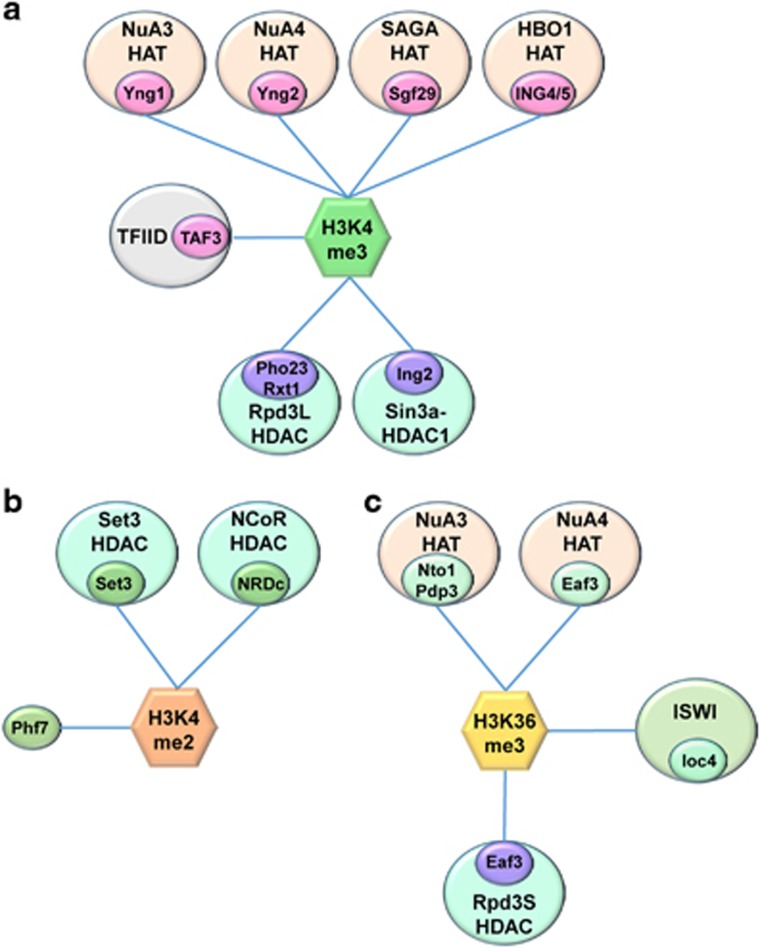
The downstream readers of co-transcriptional methylations. Multiple factors that positively and negatively affect gene expression bind co-transcriptional histone methylation of H3K4 and H3K36. (**a**) A distinct subunit (indicated by smaller circles) from multiple histone acetyltransferases (HATs) including NuA3, NuA4, SAGA and HBO1 HATs and TFIID complex binds H3K4me3. In addition, histone deacetylases (HDACs), Rpd3L in yeast and Sin3a-HDAC1 in mammals also interact with H3K4me3 via their specific subunits. (**b**) Factors that influence transcription bind to H3K4me2. The Set3 PHD finger and NRDc associated with HDACs preferentially bind H3K4me2. (**c**) Chromatin regulators that interact with H3K36me3. Two PHD finger subunits, Nto1 and Pdp3 of NuA3 HAT and Eaf3 chromodomain of both NuA4 HAT and Rpd3S HDAC interact with H3K36me3. The PWWP domain of Ioc4, a subunit of chromatin remodeler, ISW1 also binds H3K36me3.

The interaction between Set1-COMPASS and RNA polII and the correlation between H3K4me3 and transcription rate suggest a positive role of this methyl mark in transcription.^15, 16, 17, 19, 20^ Many proteins that stimulate RNA polII transcription can bind H3K4me3. In budding yeast, Yng1, Yng2 and Sgf29 are associated with distinct HATs, and these were shown to strongly bind H3K4me3.^39, 40^ Yng1 and Yng2 are PHD proteins and the yeast homologs of the ING (INhibitor of Growth) gene family that may function as tumor suppressors.^41^ Yng1 is a component of NuA3 HAT, which preferentially acetylates histone H3 at K14.^42, 43, 44^ Yng2 is a subunit of NuA4, a major HAT for histone H4.^45^ The Sgf29 tudor domain protein promotes chromatin binding of SAGA HAT and histone H3 acetylation (Figure 2a).^40^ In higher eukaryotes, the TAF3 subunit of TFIID has a PHD finger that directly binds H3K4me3 to promote RNA polII initiation.^46^ Furthermore, the interaction between H3K4me3 and ING4/ING5 PHD fingers promotes histone H3 acetylation by HBO1 HAT (Figure 2a).^47, 48^ Interestingly, some PHD finger proteins associated with transcriptional corepressors also bind H3K4me3. In yeast, Pho23 and Rxt1, both components of Rpd3 large(Rpd3L) HDAC, directly bind to H3K4me3 via their PHD fingers.^39, 49, 50, 51^ Mammalian ING2, a mSin3a-HDAC1 complex subunit also preferentially binds to H3K4me3 to promote gene repression (Figure 2a).^52^

The H3K4me2 peak in 5′ transcribed regions can be recognized by several factors, including Set3, Phf7 and nardilysin, that regulate transcription and histone acetylation (Figure 2b).^6, 53, 54^ Set3 is a component of Set3 HDAC, which also contains two deactylase subunits, Hos2 and Hst1.^55^ The Set3 PHD finger preferentially binds H3K4me2 and targets Set3 HDAC to 5′ transcribed regions.^6^

H3K36 methylation by Set2 also creates a binding site for various factors involved in histone acetylation/deacetylation and transcription elongation (Figure 2c). In yeast, the Eaf3 chromodomain, Nto1 PHD finger, Pdp3 PWWP domain and Ioc4 PWWP domain are known to bind H3K36me3.^39, 50, 51, 56, 57^ Eaf3 is found in both Rpd3 small(Rpd3S) HDAC and NuA4 HAT.^50, 51, 58^ Both Not1 and Pdp3 are components of NuA3 HAT.^39, 56^ These findings suggest both a positive and negative role of H3K36me3 in histone acetylation. The interaction between Ioc4 PWWP domain and H3K36me3 helps maintain chromatin integrity during RNA polII elongation (Figure 2c).^57^

Both H3K4 and H3K36 co-transcriptional methylations may also inhibit binding of some factors to histones or transcribed regions. BHC80 PHD finger binds unmethylated H3K4 and this binding is dramatically inhibited by H3K4 methylation.^59^ Crosslinking of histone chaperones Spt6 and Asf1 to coding regions is inhibited by H3K36 methylation.^60^ The functions of each individual downstream effector for H3K4 or H3K36 methylation remain to be completely understood.

## Deacetylation by H3K4me2-Set3 HDAC pathway delays gene induction

**Figure 3:**
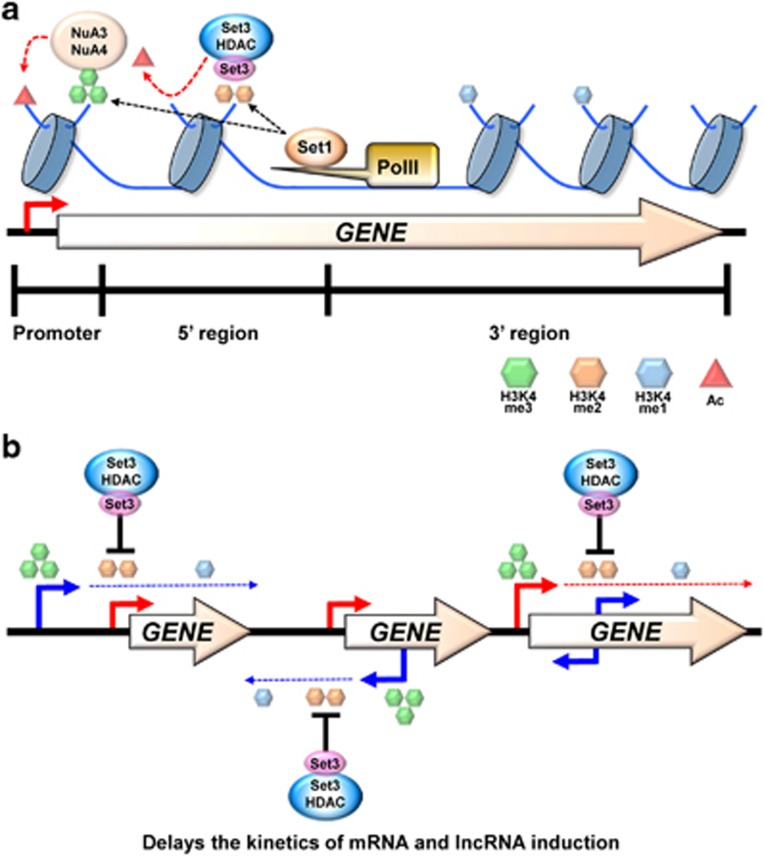
Model for regulation of histone acetylation and gene induction by Set3 HDAC. (**a**) The C-terminal domain (CTD)-interacting Set1 HMT deposits H3K4me3 and H3K4me2 to promoters and 5′ transcribed regions, respectively. NuA3 and NuA4 HATs may acetylate histones at promoters via the interaction between PHD finger proteins, Yng1 and Yng2 and H3K4me3. In 5′ transcribed regions, the Set3 PHD finger binds H3K4me2 and two histone deacetylases (HDACs), Hos2 and Hst1 deacetylate histones. (**b**) Long noncoding RNA (lncRNA) transcription from an upstream or an antisense promoter targets H3K4me2 and Set3 HDAC to messenger RNA (mRNA) promoters. Deacetylation by Set3 HDAC delays gene induction upon environmental changes. Transcription from the mRNA promoter of a gene places H3K4me2 to 5′ transcribed regions and Set3 HDAC slows the kinetics of lncRNA induction.

The Set3 HDAC consists of seven polypeptides including two histone deacetylases, Hos2 and Hst1.^55^ The Set3 protein has both a SET domain and a PHD finger.^55^ Whereas the function of the Set3 SET domain needs to be determined, the Set3 PHD finger has been shown to preferentially bind H3K4me2 peaking from 5′ to the middle parts of genes.^6, 39^ Loss of Set3 HDAC increased acetylation in 5′ transcribed regions (Figure 3a).^6^ Elimination of H3K4me2 and H3K4me3 by deleting *SET1* or Rad6-Bre1 H2B ubiquitination pathway also resulted in hyperacetylation in 5′ regions.^6^ Furthermore, mutation in the Set3 PHD finger that abrogates histone binding also increased acetylation, indicating that the interaction between H3K4me2 and the Set3 PHD finger maintains hypoacetylation in 5′ transcribed regions.^6^ Deacetylation by Set3 HDAC may suppress spreading of acetylation into gene bodies.

Interestingly, although loss of Set3 HDAC significantly changed histone acetylation, no difference in transcript levels was observed between cells containing or lacking Set3 under steady-state growth conditions.^6^ A genome-wide transcript profiling of cells grown in synthetic complete media containing glucose also revealed no detectable changes in global transcript levels upon deletion of Set3 HDAC.^8^ Interestingly, the effects of Set3 HDAC only became apparent when gene expression is dynamically changed by altering growth conditions. Histone deacetylation is generally associated with gene repression, but Set3 HDAC seems to be both repressive and activating. Set3 HDAC is required for proper induction kinetics of *GAL1* and *INO1* genes.^6, 61^ Also, this complex negatively regulates the induction of meiotic genes upon nitrogen starvation.^55^ Molecular mechanisms for how Set3 HDAC differentially controls these genes have been partially understood.

We recently showed that Set3 HDAC delays the induction of ~120 genes during carbon source shifts.^62^ Steady-state levels of transcripts were almost the same in wild type and a mutant for Set3. However, a stronger or more rapid induction of these target genes was seen in *SET3* deletion cells during carbon source shifts (Figure 3b).^12, 62^ Two histone deacetylases, Hos2 and Hst1 were also required for Set3-mediated delay of gene induction.^62^ Surprisingly, although Set3 HDAC preferentially affects histone acetylation in 5′ regions of mRNA genes where H3K4me2 peaks, Set3-repressed genes showed increased acetylation at promoter regions in *SET3* deletion cells.^6, 62^ These results suggest that the Set3-affected genes may have non-canonical patterns of histone methylation. Analysis of histone methylation pattern revealed that ~53% of Set3 target genes had high levels of H3K4me2 at transcription start sites (Figure 3b).^62^ Apparently, this pattern of H3K4me2 was established by overlapping lncRNA transcription. A majority of Set3-regulated genes were overlapped by a lncRNA transcription either from a distal or an antisense promoter.^62^ These lncRNA promoters were enriched with H3K4me3 but the associated Set3-regulated mRNA promoters had high levels of H3K4me2 (Figure 3b). At these genes, Set3 HDAC affected histone acetylation only at mRNA promoters but not at lncRNA promoters. These findings suggest that targeting of H3K4me2 and Set3 HDAC to mRNA promoters via lncRNA transcription delays or fine tunes gene induction upon environmental changes.

An additional function of H3K4me2-Set3 HDAC is to repress lncRNA transcription from cryptic promoters within 5′ transcribed regions. Although deletion of *SET3* had no effect on mRNA transcription at these genes, the overlapped cryptic promoters were much more active in this mutant during carbon source shifts (Figure 3b). Therefore, Set3 HDAC maintains optimal expression dynamics of both mRNA and lncRNA.

## The Set2-Rpd3S HDAC pathway slows mRNA and lncRNA induction

H3K36 methylation by Set2 plays important functions during transcription elongation. Although a direct binding of Set2 to elongating RNA polII and the correlation of H3K36me3 with transcription rate suggested a positive function in RNA polII transcription, loss of Set2 showed phenotypes associated with transcription inhibition. Deletion of *SET2* or mutation of H3K36 to A or R bypassed loss of Bur1, a positive elongation factor.^50, 63^ Mutations of other elongation factors such as Spt16 and Spt5 were also suppressed by loss of Set2.^64, 65^ Furthermore, although many elongation mutants are sensitive to 6-AU or MPA that inhibits elongation, *SET2* deleting cells are resistant to these chemicals.^30, 50^

**Figure 4:**
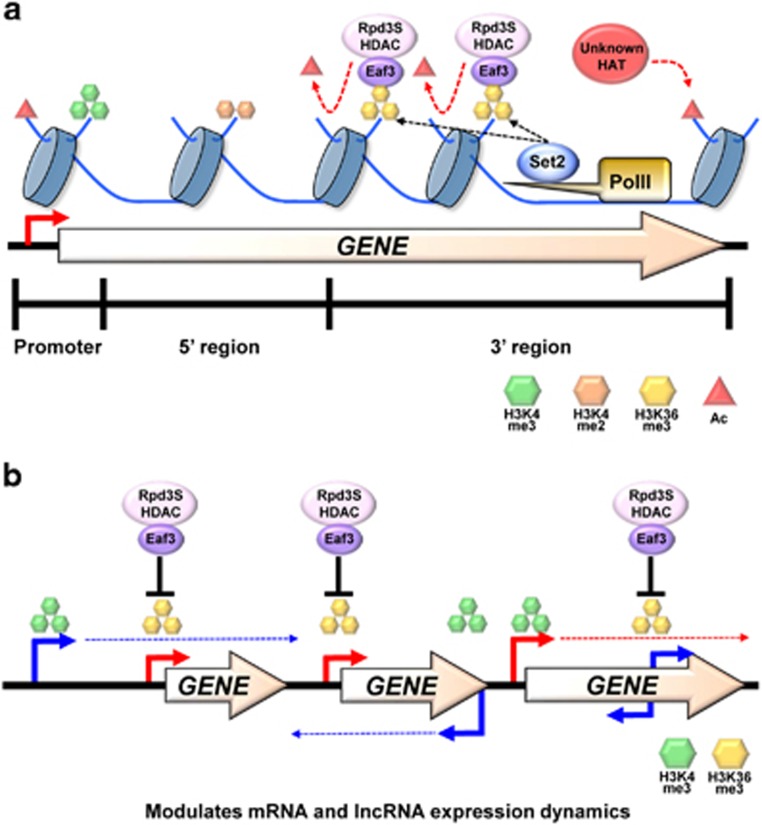
Model for modulation of acetylation and gene induction by the Set2-Rpd3S pathway. (**a**) Set2 HMT interacts with elongating RNA polII and methylates on H3K36 in 3′ transcribed regions. Eaf3 chromodomain binds H3K36me2/3 and Rco1 PHD finger interacts with histone tails to stabilize chromatin binding of Rpd3S. Rpd3 deacetylates histones to slow elongation and suppress cryptic promoters. (**b**) lncRNA transcription from an upstream or an antisense promoter targets H3K36me3 and Rpd3S HDAC to mRNA promoters. Deacetylation at mRNA promoters by Rpd3S slows gene induction. Transcription from the mRNA promoter of a gene localizes H3K36me3 to inducible cryptic promoters. Deacetylation by Rpd3S histone deacetylase (HDAC) delays the rate of lncRNA induction. HMT, histone methyltransferase; lncRNA, long noncoding RNA; mRNA, messenger RNA; RNA polII, RNA polymerase II.

During transcription elongation, histones are thought to be acetylated by one or more HATs to promote elongation on the chromatin template. Once RNA polII passes through the gene, deacetylation is important to re-establish a repressive chromatin configuration that blocks aberrant transcription. Set2-mediated H3K36 methylation facilitates histone deacetylation by Rpd3S within coding regions (Figure 4a).^50, 51, 58, 66, 67^ Two subunits, Eaf3 and Rco1, appear to be important for chromatin binding of this complex. Eaf3 chromodomain directly binds to methylated histones on H3K36 and Rco1 PHD finger interacts with unmodified histone tails.^39, 68, 69, 70^ Rpd3S seems to be also recruited to transcribed regions by elongating RNA polII via interaction with the phosphorylated CTD, but histone deacetylation requires a direct interaction of this complex with methylated histones on H3K36.^66, 67^ Deacetylation by Rpd3S within coding regions slows RNA polII elongation (Figure 4a).^50^ The Set2-Rpd3S pathway also contributes to repress cryptic promoters that produce sense or antisense lncRNAs. Many transcription elongation factors and chromatin regulators including Spt6, Spt16 and Ctk1 suppress internal initiation from cryptic promoters within open reading frames.^71^ Loss of the Set2-Rpd3S pathway resulted in hyperacetylation within coding regions and initiation of RNA polII transcription from cryptic promoters.^51, 72^ A recent study also identified a novel class of antisense transcripts repressed by Set2.^73^

Although previous studies on the Set2-Rpd3S pathway elucidated molecular mechanisms for how the pathway is targeted and inhibits lncRNA transcription from cryptic promoters, its function in mRNA gene regulation remains unclear. Surprisingly, despite its importance in suppression of cryptic promoters via histone deacetylation, global mRNA transcript levels were not affected by loss of this pathway under a steady-state growth condition.^8^ To further explore the exact function of the Set2-Rpd3S pathway, we recently monitored genome-wide transcript levels in wild type and *SET2* deletion cells by high-resolution, strand-specific tiling arrays during carbon source shifts.^74^ Although steady-state levels of transcripts were not affected, ~80 genes showed a strong and more rapid induction upon *SET2* deletion.^74^ These findings suggest that Set2-Rpd3S pathway is important for modulating mRNA expression dynamics during environmental changes (Figure 4b).

Analysis of Set2-affected genes revealed that 69% of these genes had an overlapping lncRNA transcription from either an upstream or an antisense promoter.^74^ One class of Set2-repressed genes was overlapped with lncRNA transcription from a distal promoter (Figure 4b). *AAD10* is one of these Set2-repressed genes, and is overlapped by sense lncRNA transcription from the promoter of upstream gene *YJR154W*. Although *AAD10* lncRNA was detected in rich media, neither Aad10 nor Yjr154w protein was observed, suggesting that this lncRNA may not be translated into a protein. This locus showed an interesting pattern of co-transcriptional histone methylation. Although H3K4me3 and H3K36me3 are in general enriched at promoters and 3′ part of genes, respectively, the *AAD10* promoter showed high levels of H3K36me3 but not H3K4me3 (Figure 4b).^74^

The overlapping transcription at the majority of Set2-regulated genes came from downstream antisense promoters (Figure 4b). For example, the *SUL1* gene was overlapped by the antisense lncRNA SUT452, which is constitutively transcribed. H3K4me3 was highest at SUT452 promoter near the 3′ end of *SUL1,* while the *SUL1* mRNA promoter had high levels of H3K36me3.^74^ We revealed that H3K36me3 deposited during lncRNA transcription facilitated histone deacetylation of the overlapped mRNA promoter by Rpd3S.^74^ Loss of Set2 or subunits of Rpd3S showed increased acetylation at *AAD10* and *SUL1* promoters but not at *YJR154W* or SUT452 promoters. Accordingly, *AAD10* and *SUL1* transcripts but not the associated lncRNAs were strongly induced in mutants for the Set2-Rpd3S pathway during carbon source shifts.^74^

A previous study reported that although 1685 genes showed increased acetylation within open reading frames in *SET2* deleting cells, only half of them produced internal lncRNA transcripts.^72^ It is possible that any lncRNA transcripts produced from these genes are not observed because they can be rapidly degraded by nucleases such as the nuclear exosome. For example, both antisense and short sense transcripts were seen at *STE11* and *CTT1* in double mutants lacking both Rpd3S (rco1Δ) and nuclear exosome (rrp6Δ), but only the short sense transcripts were seen upon deletion of *RCO1* alone.^75^ These suggest that antisense transcripts from *STE11* and *CTT1* are rapidly degraded by nuclear exosome.

Another interesting possibility is that cryptic promoters within genes may be inducible and therefore active only under certain conditions. Previous studies revealed that some cryptic transcripts were induced by nutritional shifts.^62, 71^ We identified 118 internal cryptic promoters generating a short sense transcript and 639 producing an antisense transcript in *SET2* deletion cells during carbon source shifts.^74^ Surprisingly, 416 of them were induced by specific carbon sources (Figure 4b). Furthermore, three cryptic promoters in the *PCA1* gene that are repressed by Set2 differently responded to nutritional shifts.^74^ One was constitutively active but the other two were induced or repressed during carbon source shifts. These results suggest that in addition to mRNA genes, the Set2-Rpd3S pathway can delay the induction of lncRNAs upon environmental changes.

## Gene regulation by overlapping lncRNA transcription

Two recent studies from our group have revealed that H3K4me2-Set3 HDAC and the Set2-Rpd3S pathway modulate the induction of mostly distinct target genes.^62, 74^ However, the genes regulated by both pathways have a common feature. A majority of them are overlapped by lncRNA transcription either from a distal or an antisense promoter. The overlapping lncRNA transcription, rather than the lncRNA itself, may target H3K4me2 or H3K36me3 to promoter regions of distinct genes and delay the kinetics of gene induction.

**Figure 5:**
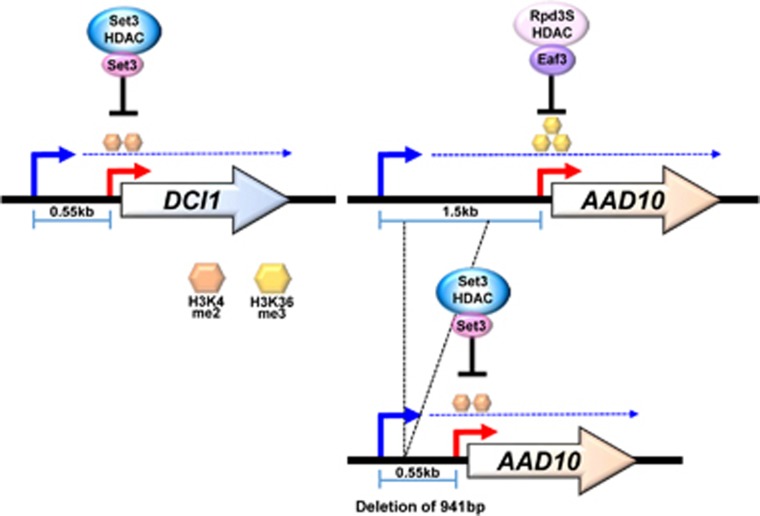
Model for regulation of gene induction by lncRNA transcription and two HDACs. Set3 HDAC and the Set2-Rpd3S pathway negatively regulate the kinetics of *AAD10* and *DCI1* induction, respectively. H3K4me2 and Set3 HDAC are targeted to *DCI1* mRNA promoter via an overlapping lncRNA transcription and slow *DCI1* induction. In contrast, lncRNA transcription from an upstream promoter of *AAD10* targets H3K36me3 and Rpd3S to *AAD10* mRNA promoter. Surprisingly, shortening the distance between the two promoters of *AAD10* replaces H3K36me3-Rpd3S with H3K4me2-Set3 HDAC for *AAD10* repression. Thus, position of the lncRNA promoter may be important for specifying the repressive effects of the two HDACs. HDAC, histone deacetylase; mRNA, messenger RNA; lncRNA, long noncoding RNA.

*DCI1* is one of the Set3-repressed genes overlapped by lncRNA transcription from a distal promoter (Figure 5). Upon *SET3* deletion, lncRNA transcript levels were not changed, but *DCI1* mRNA transcripts were elevated during galactose incubation. Before induction, the *DCI1* mRNA promoter had a peak of H3K4me2 as a result of the overlapping lncRNA transcription. The *DCI1* promoter showed increased acetylation upon loss of Set3, explaining its elevated transcription upon induction.^62^ No derepression of this gene was observed in mutants for the Set2-Rpd3S pathway. In contrast, *AAD10* is also overlapped with lncRNA transcription from an upstream promoter, but is repressed by Set2 (Figure 5). Interestingly, the *AAD10* promoter was enriched with H3K36me3 and derepressed in mutants for the Set2-Rpd3S pathway but not Set3C.

What determines the specificity for these two pathways? An interesting difference between Set3- and Set2-repressed genes is the distance between mRNA and lncRNA promoters (Figure 5). For example, the lncRNA and mRNA promoters of the *DCI1* are ~0.55 kb apart, but the distance between the two *AAD10* promoters is ~1.5 kb. Furthermore, the average distances between the lncRNA and mRNA promoters of Set3-repressed and Set2-regulated genes were ~0.9 kb and 2.0 kb, respectively. These findings support the idea that the distance between mRNA and lncRNA promoter may specify the Set2-Rpd3S or Set3 HDAC-mediated repression.^74^

Consistent with this, reducing the distance between the two promoters of *AAD10* caused expression to become insensitive to the Set2-Rpd3S pathway. H3K36me3 was significantly reduced at the *AAD10* mRNA promoter and histone acetylation was not changed in mutants for the Set2-Rpd3S pathway. In addition, *AAD10* transcript levels were very similar in both wild type and *SET2* deletion cells.^74^ These results indicate that shortening the distance between the two promoters of *AAD10* alleviates repression by the Set2-Rpd3S pathway. Furthermore, although H3K4me2 was low at *AAD10* promoter in the normal context, an elevated level was seen when the distance was reduced. Correspondingly, histone acetylation and *AAD10* transcript levels were now increased in mutants for Set3 HDAC. Thus, alteration of the distance between the two promoters switches the repression of *AAD10* by H3K36me3-Rpd3S to the H3K4me2-Set3 HDAC repression pathway.

Transcription of lncRNA as well as lncRNA itself can play important roles in gene regulation.^13^ Our recent findings suggest that although lncRNA can be transcribed from any site of a genome, positions of overlapping lncRNA promoters may be important for targeting distinct histone methylations and chromatin regulators to promoter regions of an associated mRNA gene.

## Discussion

Co-transcriptional histone methylations and their downstream effectors have critical roles in almost all steps of gene regulation including initiation, elongation, RNA processing and termination. Despite their importance, global gene expression is often not significantly affected by loss of these factors in optimized or laboratory growth conditions. Instead, stronger effects can be observed in more realistically dynamic conditions where gene expression is continuously changing. H3K4me2-Set3 HDAC and the Set2-Rpd3S pathways deacetylate histones at certain mRNA or lncRNA promoters where H3K4me2 and H3K36me3 are enriched, respectively, and negatively regulate the rate of mRNA or lncRNA induction.^62, 74^ However, the HATs responsible for acetylation at these regions are not completely known. Some factors associated with distinct HATs have been shown to bind H3K36me3. For example, Nto1 and Pdp3 of NuA3 preferentially bind to H3K36me3.^39, 56^ Furthermore, Eaf3 is a component of both NuA4 HAT and Rpd3S HDAC. Future works will determine if NuA3 or NuA4 HAT can acetylate histones at Set2-repressed promoters and increase the rate of mRNA and lncRNA induction upon environmental changes.

Regulation of gene expression dynamics is likely to be essential for normal development process, adaptation to new environmental conditions and proper cellular responses to extracellular stresses. A previous report revealed that knockdown of many chromatin regulators had no obvious effect on stem cell maintenance.^76^ Instead, greater effects are observed upon loss of chromatin regulators when cells are triggered with stresses or undergo cellular differentiation, suggesting that the maintenance of gene expression dynamics during this period is critical for fate decisions of stem cells. Future works need to identify the genes that are dynamically regulated during stem cell differentiation, and study the function of chromatin regulators in this process.

The factors involved in co-transcriptional histone methylations and their downstream effectors have been directly linked to many cancers.^77, 78, 79^ Altered expression, translocation, and amplification of genes for H3K4 HMTs and H3K36 HMTs have been reported in distinct types of cancers.^78, 79^ Overexpression or mutations of genes encoding HDMs for both methyl marks are also observed in cancers.^77, 79^ In addition, ING family proteins that directly bind methylated lysines function as tumor suppressors or oncogenes.^48, 80^ Current studies tend to directly compare global gene expression in normal and cancer cells to identify cancer-associated genes. However, since mutation or overexpression of the factors related to co-transcriptional methylations will have greater effects on gene expression changes in a more dynamic condition, studying their functions during cancer development and progression will be much more important and allow us to identify novel genes that are differentially expressed in cancer cells.
